# Nesfatin-1 across human reproduction and early life: implications for infertility, pregnancy complications, and lactation

**DOI:** 10.3389/fendo.2026.1796327

**Published:** 2026-05-11

**Authors:** Regina Katona, Dora Filarszky, Simone Funke, Reka Anna Vass

**Affiliations:** 1 Department of Obstetrics and Gynecology, Medical School, University of Pécs, Pécs, Hungary; 2 National Laboratory on Human Reproduction, University of Pécs, Pécs, Hungary

**Keywords:** biomarker, developmental programming, gestational diabetes, immunometabolism, maternal–fetal interface, NUCB2

## Abstract

Nesfatin-1, a peptide derived from nucleobindin-2 (NUCB2), is a widely expressed neuroendocrine factor originally identified for its anorexigenic effects in the central nervous system. Beyond energy homeostasis, accumulating evidence implicates nesfatin-1 in the regulation of glucose metabolism, stress-axis activity, inflammation, and reproductive function. These properties make nesfatin-1 particularly relevant to human reproduction and early life, physiological states characterized by profound metabolic and endocrine adaptations. The objective of this review is to examine whether nesfatin-1 functions as an integrative endocrine signal linking metabolic, stress-related, and reproductive pathways across the reproductive life course. We synthesize current human evidence on nesfatin-1 in infertility, assisted reproductive technologies, pregnancy adaptations and complications, the maternal–fetal interface, lactation, and postpartum metabolic recovery. Nesfatin-1 has been detected in several biological compartments relevant to reproductive physiology, including maternal plasma, follicular fluid, amniotic fluid, cord blood, and human breast milk. Clinical studies investigating these compartments report heterogeneous findings, with nesfatin-1 levels described as increased, decreased, or unchanged across similar clinical conditions. This variability likely reflects the context-dependent regulation of nesfatin-1 by metabolic status, stress-axis activation, inflammatory signaling, and reproductive stage. In this review, we synthesize current human evidence on nesfatin-1 across the continuum of reproduction, pregnancy, early development, and lactation, and propose a framework to reconcile these inconsistent findings. We argue that nesfatin-1 should be viewed as a context-dependent endocrine integrator reflecting the interaction of metabolic, stress-related, inflammatory, and reproductive axes rather than as a unidirectional biomarker. We further highlight key methodological challenges, including assay variability, timing of sampling, and population heterogeneity, and discuss the potential of nesfatin-1 as a longitudinal or stratification biomarker in reproductive endocrinology. Finally, we outline priorities for future research to clarify its physiological role and translational relevance in human reproduction and early life.

## 1 Introduction

Human reproduction and early life represent periods of profound endocrine, metabolic, and immunological adaptation. From preconception and fertility to pregnancy, fetal development, lactation, and postpartum recovery, successful reproductive outcomes depend on the tight integration of energy availability, stress responsiveness, inflammatory balance, and reproductive hormone signaling. Disruption of this integration contributes to infertility, suboptimal outcomes of assisted reproductive technologies (ART), pregnancy complications such as gestational diabetes mellitus (GDM) and hypertensive disorders of pregnancy, and adverse developmental programming in offspring (1, 2).

Infertility represents a major global health concern, affecting an estimated 15–20% of couples worldwide, with metabolic disorders, obesity, chronic stress, and inflammatory conditions increasingly recognized as important contributors to impaired reproductive function (3, 4). These factors influence multiple levels of reproductive physiology, including hypothalamic–pituitary–gonadal (HPG) axis regulation, ovarian steroidogenesis, follicular development, implantation, and placental function. As a result, growing attention has focused on endocrine signals that integrate metabolic and stress-related pathways with reproductive physiology (1, 2). Epidemiological analyses further emphasize the heterogeneity of infertility across populations and underscore the need to better understand the biological mechanisms linking metabolic health and reproductive outcomes (5).

Nesfatin-1, a peptide derived from the precursor nucleobindin-2 (NUCB2), was initially identified as a hypothalamic anorexigen involved in appetite suppression and energy balance (6, 7). Nesfatin-1 was originally identified as a hypothalamic peptide involved in the regulation of appetite and energy homeostasis, where it exerts anorexigenic effects and reduces food intake (6). Subsequent studies have demonstrated that nesfatin-1 also participates in the control of glucose metabolism, insulin secretion, and energy balance through both central and peripheral mechanisms (1, 2). Through these actions, nesfatin-1 functions as an integrative signal linking energy availability with neuroendocrine and metabolic regulation (2, 8). Subsequent research has demonstrated that nesfatin-1 is widely expressed in both central and peripheral tissues, including the gastrointestinal tract, pancreas, adipose tissue, pituitary gland, gonads, placenta, and mammary gland. Beyond its role in energy balance, nesfatin-1 participates in the regulation of glucose metabolism, insulin secretion, autonomic function, stress-axis activation, and inflammatory signaling (1, 8–10). Through these pleiotropic actions, nesfatin-1 has emerged as a candidate integrator linking metabolic state and stress responses with reproductive function.

Experimental studies suggest that nesfatin-1 can influence reproductive physiology at multiple levels. In the central nervous system, nesfatin-1 is expressed in hypothalamic nuclei involved in reproductive and stress regulation and interacts with gonadotropin-releasing hormone (GnRH), kisspeptin, and corticotropin-releasing hormone pathways, thereby modulating HPG axis activity (11, 12). Peripheral studies further indicate that nesfatin-1 is present in reproductive tissues, including the ovary, endometrium, and placenta, where it may influence steroidogenesis, follicular development, decidualization, trophoblast function, and local metabolic signaling (13–15). These findings suggest that nesfatin-1 may operate as a multi-level regulator linking systemic metabolic signals with reproductive physiology.

In humans, nesfatin-1 has been detected in multiple biological compartments that are highly relevant to reproduction and development, including maternal plasma, follicular fluid, amniotic fluid, cord blood, and human breast milk (16–18). Clinical studies have examined associations between circulating or compartment-specific nesfatin-1 levels and infertility, polycystic ovary syndrome, outcomes of assisted reproductive technologies, GDM, hypertensive disorders of pregnancy, fetal growth patterns, lactation, and postpartum metabolic adaptation. However, reported findings are strikingly heterogeneous, with nesfatin-1 levels described as increased, decreased, or unchanged across similar clinical conditions. Meta-analyses frequently report substantial inter-study heterogeneity, limiting definitive conclusions and clinical translation (19–21).

Rather than reflecting true biological inconsistency, these conflicting results likely arise from the context-dependent regulation of nesfatin-1 and from considerable methodological variability across human studies. Nesfatin-1 is responsive to metabolic status, inflammatory signals, stress-axis activation, sex steroids, and disease stage, all of which vary markedly across reproductive states and clinical populations. In addition, differences in assay specificity, sample type (plasma, serum, milk), timing of sampling (gestational age, postpartum period, fasting status), and preanalytical handling further complicate interpretation of human biomarker data (2, 22).

**Figure 1:**
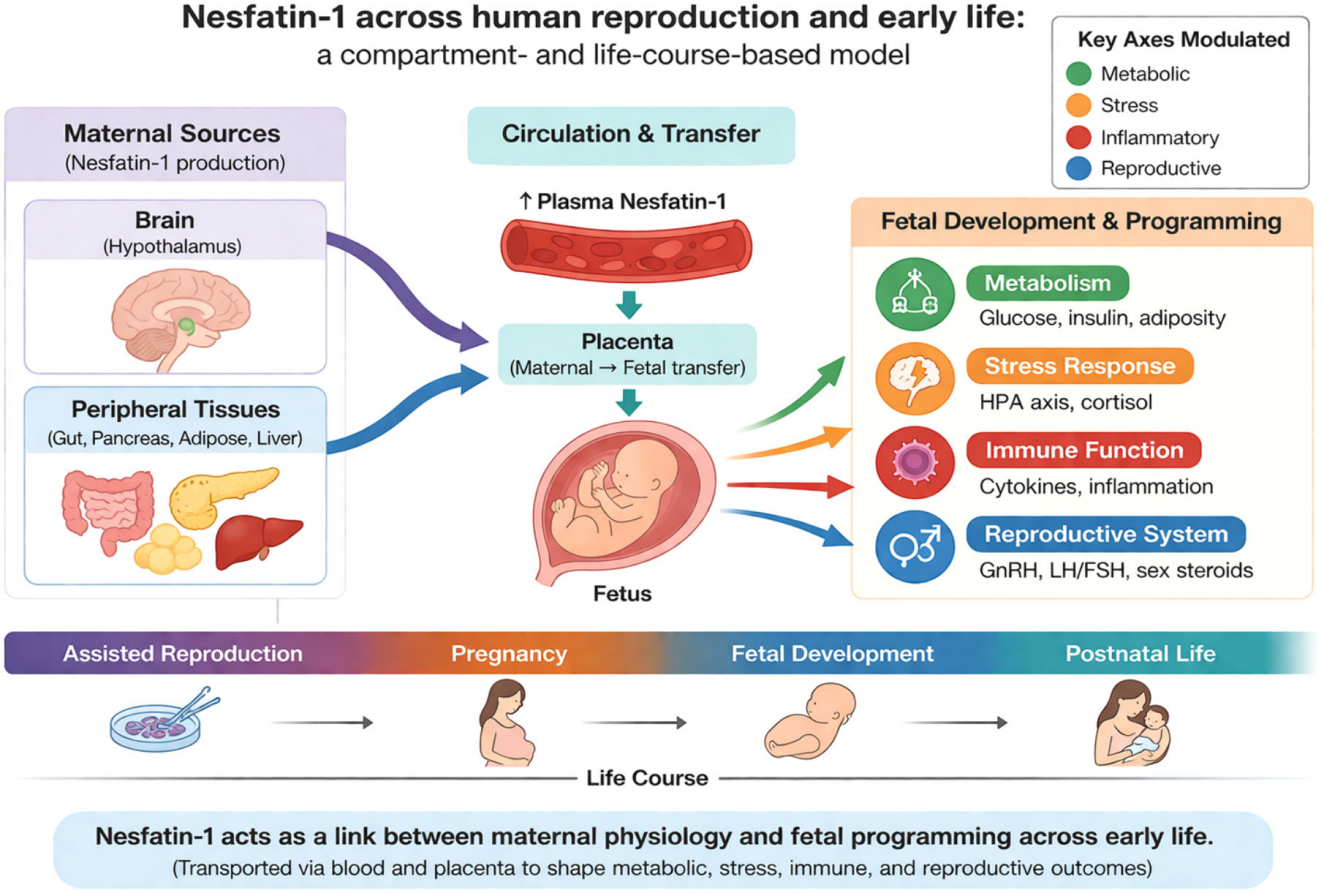
Nesfatin-1 across human reproduction and early life: a compartment- and life-course–based model. Nesfatin-1, derived from nucleobindin-2 (NUCB2), is expressed in central and peripheral tissues relevant to reproduction, pregnancy, and lactation. Central nesfatin-1 signaling in hypothalamic nuclei integrates metabolic, stress-related, and reproductive inputs and modulates hypothalamic–pituitary axes, while peripheral nesfatin-1 is produced by gastrointestinal, pancreatic, adipose, placental, and mammary tissues. During pregnancy and early life, nesfatin-1 is detectable in multiple biological compartments, including maternal plasma, amniotic fluid, cord blood, and human milk. Circulating nesfatin-1 reflects the integrated output of metabolic, inflammatory, and stress axes rather than central activity alone, contributing to heterogeneous biomarker findings across clinical conditions. This model highlights nesfatin-1 as a context-dependent endocrine integrator linking maternal metabolic state, fetal environment, and postnatal adaptation (2, 6, 8, 10–12, 16–18, 21, 23, 24). FSH, Follicle-stimulating hormone; GnRH, Gonadotropin-releasing hormone; HPA axis, Hypothalamic–pituitary–adrenal axis; LH, Luteinizing hormone; Ins, Insulin; X/A-like cells, Gastric enteroendocrine cells producing ghrelin/nesfatin.

The aim of the present review is therefore to synthesize current human evidence on nesfatin-1 across the continuum of reproduction and early life, including infertility, assisted reproductive technologies, pregnancy adaptations and complications, maternal–fetal signaling, lactation, and postpartum metabolic regulation. We further propose a compartment-based and life-course framework to reconcile heterogeneous findings by integrating biological context with methodological considerations. By framing nesfatin-1 as a context-sensitive endocrine integrator linking metabolic, stress-related, and reproductive pathways, rather than a simple unidirectional biomarker, this review aims to clarify its physiological relevance and identify priorities for future translational research in reproductive endocrinology and developmental programming (2, 6, 8, 10–12, 16–18, 21, 23, 24) (**Figure 1**).

## 2 Biology of NUCB2/nesfatin-1 relevant to reproduction

### 2.1 Tissue sources relevant to pregnancy and lactation

Nesfatin-1 is generated by post-translational processing of the NUCB2, which is widely expressed in both central and peripheral tissues. In the context of human reproduction and early life, NUCB2/nesfatin-1 expression has been documented in several organs that undergo major functional adaptation during pregnancy and lactation. Within the central nervous system, nesfatin-1 is abundantly expressed in hypothalamic nuclei involved in energy balance, stress regulation, and reproductive control, including the paraventricular, arcuate, and supraoptic nuclei (6, 11).

Peripherally, NUCB2/nesfatin-1 is produced in the gastrointestinal tract, particularly in gastric X/A-like cells, pancreatic islets, adipose tissue, pituitary, gonads, placenta, and mammary gland (8, 10, 23). During pregnancy, these tissues contribute to the profound endocrine and metabolic remodeling required to support fetal growth. Human studies have demonstrated measurable nesfatin-1 levels in maternal plasma, amniotic fluid, cord blood, and human breast milk, indicating that nesfatin-1 participates in both prenatal and postnatal biological compartments relevant to offspring development (16–18).

The presence of nesfatin-1 in human milk, including colostrum and mature milk, suggests a potential role in postnatal metabolic signaling and appetite regulation, while amniotic fluid nesfatin-1 may reflect intrauterine metabolic or inflammatory conditions rather than direct fetal secretion alone. However, the relative contribution of maternal, placental, and fetal sources to nesfatin-1 levels in these compartments remains incompletely defined.

### 2.1.Central versus peripheral signaling: why blood does not equal brain

A critical aspect of nesfatin-1 biology relevant to biomarker interpretation is the dissociation between its central and peripheral actions. Nesfatin-1 was originally characterized as a centrally acting neuropeptide, with intracerebroventricular administration producing robust anorexigenic and stress-related effects independent of circulating levels (1, 6, 7). Central nesfatin-1 signaling influences hypothalamic-pituitary-gonadal and hypothalamic-pituitary-adrenal axis activity, autonomic output, and neuroendocrine stress responses, all of which are highly relevant during pregnancy and postpartum adaptation (12).

In contrast, circulating nesfatin-1 reflects the integrated output of multiple peripheral tissues and does not necessarily mirror hypothalamic activity. The extent to which peripheral nesfatin-1 crosses the blood–brain barrier remains uncertain, and current evidence suggests that central and peripheral nesfatin-1 pools are at least partially independent (2, 23). This central–peripheral dissociation is particularly important in pregnancy and lactation, where physiological stress, inflammation, insulin resistance, and hormonal fluctuations may differentially regulate tissue-specific nesfatin-1 expression.

Consequently, circulating nesfatin-1 levels should be interpreted as indicators of systemic metabolic–endocrine context rather than direct proxies of central neuroendocrine signaling.

### 2.2 Unknown receptor identity

Despite extensive investigation, the cognate receptor for nesfatin-1 has not yet been definitively identified. Pharmacological and signaling studies support the existence of a Gi protein–coupled receptor mediating nesfatin-1 actions, with downstream activation of pathways including AMP-activated protein kinase (AMPK), mitogen-activated protein kinase (MAPK), and mechanistic target of rapamycin complex 1 *(*mTORC1) in a tissue-specific manner (25, 26). However, the absence of receptor de-orphanization limits precise interpretation of dose–response relationships and hampers the distinction between direct and indirect effects.

This knowledge gap has important consequences for clinical biomarker studies (2, 23, 25). Without a defined receptor, it remains unclear whether measured circulating nesfatin-1 reflects bioactive signaling capacity, compensatory responses, or altered peptide processing (2, 8, 23). This limitation is particularly relevant in reproductive and early-life studies, where physiological states such as pregnancy, lactation, and postpartum recovery involve rapid and dynamic endocrine changes (2, 22).

Accordingly, nesfatin-1 should currently be regarded as a context-dependent endocrine indicator rather than a unidirectional biomarker. Interpretation of nesfatin-1 levels in maternal plasma, amniotic fluid, or breast milk requires careful consideration of biological compartment, reproductive stage, metabolic status, and methodological factors, a theme that underpins the subsequent sections of this review (2, 17, 18).

## 3 Preconception and infertility

Female fertility is tightly coupled to metabolic status and neuroendocrine regulation, and disruption of this integration is a major contributor to anovulation, subfertility, and infertility. Energy imbalance, insulin resistance, chronic inflammation, and stress-axis activation influence reproductive function at multiple levels, including hypothalamic–pituitary–gonadal (HPG) axis signaling, gonadotropin secretion, ovarian steroidogenesis, and follicular development (8, 11, 12). These interconnected pathways underscore the importance of endocrine signals that integrate metabolic and stress-related cues with reproductive physiology.

Nesfatin-1, a peptide derived from nucleobindin-2 (NUCB2), has emerged as a candidate mediator within this integrative network. Initially identified as a hypothalamic regulator of appetite and energy homeostasis (6), nesfatin-1 is now known to be widely expressed in both central and peripheral tissues, including the hypothalamus, pancreas, adipose tissue, and reproductive organs (1, 23). Beyond its role in energy balance, nesfatin-1 participates in the regulation of glucose metabolism, insulin secretion, stress-axis activation, and inflammatory signaling, positioning it as a potential link between systemic metabolic status and reproductive function (2, 9).

Experimental evidence suggests that nesfatin-1 may influence reproductive competence through both central and peripheral mechanisms. Within the central nervous system, nesfatin-1 is expressed in hypothalamic nuclei involved in reproductive and stress regulation and modulates gonadotropin-releasing hormone (GnRH) and luteinizing hormone (LH) secretion, likely through interactions with kisspeptin and corticotropin-releasing hormone pathways (11, 12, 27). These findings support the concept that nesfatin-1 may act as a metabolic gatekeeper of reproduction, restraining reproductive axis activity under conditions of metabolic imbalance or stress (2, 8, 11). At the peripheral level, nesfatin-1 expression in ovarian tissue suggests potential roles in follicular development and steroidogenesis through local autocrine or paracrine mechanisms (14, 28).

Human studies investigating nesfatin-1 in infertility and related conditions, particularly polycystic ovary syndrome (PCOS), report heterogeneous findings. Circulating nesfatin-1 levels have been described as increased, decreased, or unchanged in women with PCOS compared with controls (19, 20). Rather than reflecting methodological inconsistency alone, this variability likely reflects the context-dependent regulation of nesfatin-1. PCOS is a heterogeneous condition characterized by varying degrees of insulin resistance, obesity, hyperandrogenism, and inflammatory activation, all of which may influence nesfatin-1 expression and secretion (19, 29). Differences in metabolic phenotype, particularly lean versus obese PCOS, as well as study design and treatment exposure, are therefore likely to contribute to divergent observations.

Taken together, current evidence supports a model in which nesfatin-1 does not act as a direct determinant of fertility but rather as an integrative signal reflecting interactions between metabolic status, stress responsiveness, and reproductive function. Altered nesfatin-1 levels in infertile women may therefore represent adaptive or compensatory neuroendocrine responses to metabolic imbalance or chronic stress, rather than a primary pathogenic mechanism (12, 23). This context dependence likely underlies the inconsistent associations reported in human studies and limits the utility of nesfatin-1 as a standalone biomarker of infertility (5, 19, 20).

From a translational perspective, nesfatin-1 may be most informative when assessed longitudinally or in combination with established metabolic and reproductive markers. Such an approach may help identify subgroups of women with distinct metabolic or stress-related profiles within broader infertility diagnoses. These considerations provide a rationale for examining nesfatin-1 in more complex clinical settings, including assisted reproductive technologies, where metabolic, endocrine, and psychological factors converge to influence reproductive outcomes.

### 3.1 Central regulation of reproductive function

Experimental studies demonstrate that nesfatin-1 participates in the central regulation of the HPG axis. NUCB2/nesfatin-1 is expressed in hypothalamic nuclei critical for reproductive control, including the paraventricular and arcuate nuclei, where it interacts with GnRH, kisspeptin, and corticotropin-releasing hormone signaling pathways (11, 12, 27). Central administration of nesfatin-1 suppresses LH secretion in animal models, supporting an inhibitory role under conditions of negative energy balance or physiological stress (9). These findings reinforce the concept that nesfatin-1 functions as a metabolic gatekeeper, linking energy availability and stress signaling to reproductive axis activity (2, 8, 11).

Although direct assessment of central nesfatin-1 signaling in humans is not feasible, circulating nesfatin-1 levels may reflect alterations in this central–peripheral neuroendocrine network. In metabolically challenged states, including obesity and insulin resistance, changes in circulating nesfatin-1 may therefore represent downstream manifestations of altered hypothalamic regulation rather than isolated peripheral phenomena (2, 12, 19, 23).

### 3.2 Peripheral actions and metabolic infertility

In addition to central effects, nesfatin-1 is expressed in peripheral reproductive tissues, including the ovary, where it modulates steroid hormone production, cellular proliferation, and apoptosis in granulosa and luteal cells (14, 15). These findings suggest that nesfatin-1 may influence follicular development and ovarian function through local regulatory mechanisms, complementing central control of gonadotropin secretion (28).

In humans, altered circulating nesfatin-1 levels have been reported in metabolic conditions associated with infertility, most notably PCOS. Several studies describe associations between nesfatin-1 and body mass index, insulin resistance, androgen excess, and gonadotropin imbalance, although the direction and magnitude of these associations vary considerably across populations (19, 20). Meta-analyses further highlight substantial heterogeneity, suggesting that nesfatin-1 levels are influenced by obesity status, metabolic phenotype, ethnicity, and treatment exposure rather than reflecting a uniform disease-specific signal (19, 29).

### 3.3 Interpretation in the preconception context

Collectively, available evidence supports a role for nesfatin-1 as a mediator linking metabolic and stress-related cues to reproductive function, rather than as a direct regulator of fertility (2, 8, 11). In the preconception setting, altered nesfatin-1 levels are likely to reflect adaptive or compensatory endocrine responses to metabolic imbalance or chronic stress (12, 23). This context dependence provides a plausible explanation for the inconsistent findings reported across clinical studies and underscores the limitations of interpreting nesfatin-1 as a standalone infertility biomarker (2, 19, 20).

**Figure 2:**
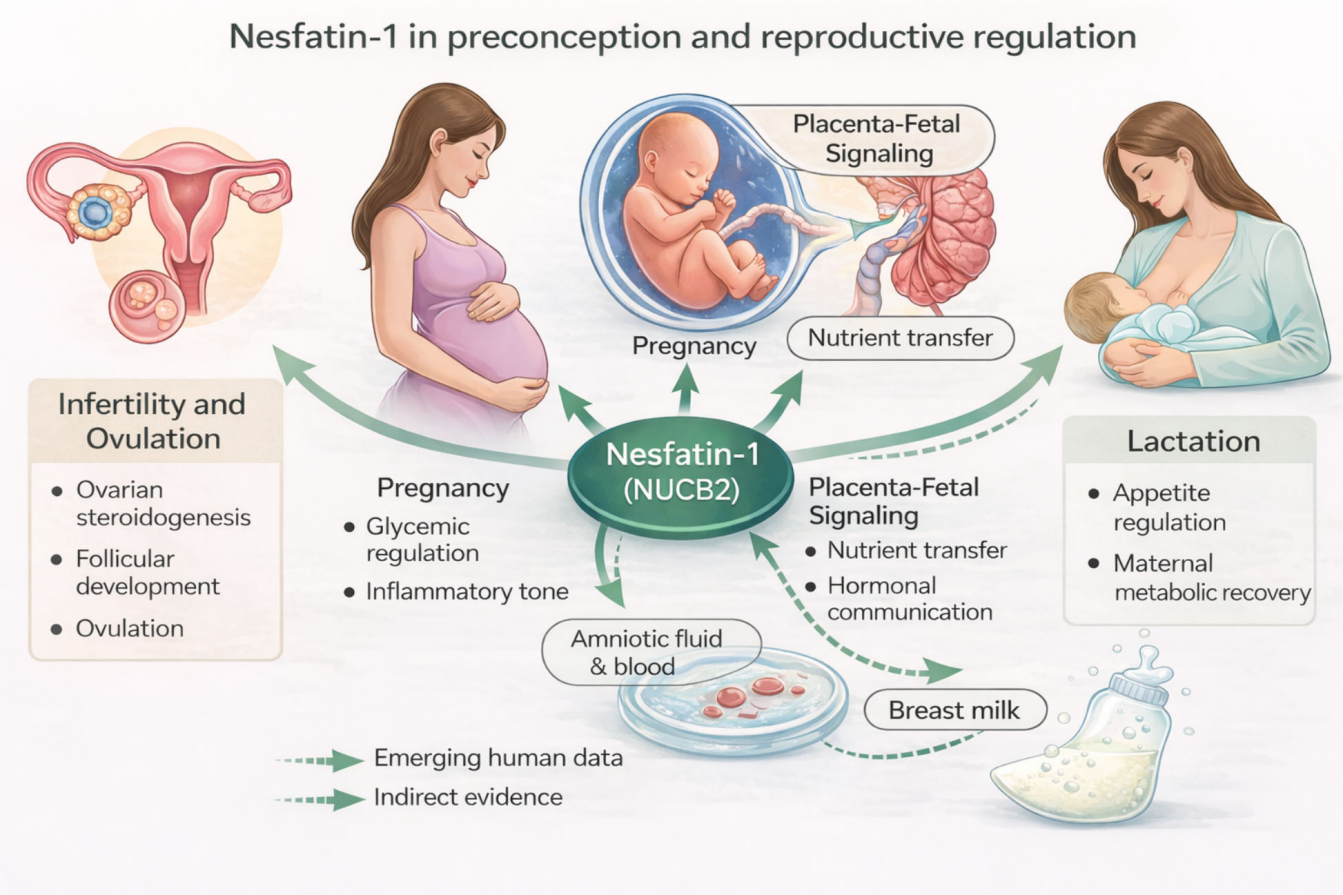
Nesfatin-1 in preconception and reproductive regulation. Schematic overview of the proposed roles of nesfatin-1 (derived from nucleobindin-2, NUCB2) in preconception physiology and female reproductive function. Nesfatin-1 is expressed in central hypothalamic nuclei involved in hypothalamic–pituitary–gonadal (HPG) axis regulation, where it interacts with gonadotropin-releasing hormone and stress-related signaling pathways. In the periphery, nesfatin-1 is detectable in ovarian tissue and may influence follicular development, steroidogenesis, and ovulatory processes through autocrine or paracrine mechanisms. Circulating nesfatin-1 reflects integrated metabolic, inflammatory, and stress-related inputs rather than direct central signaling. Solid arrows indicate mechanisms supported by experimental or human evidence, whereas dashed arrows represent indirect or context-dependent associations. The figure emphasizes nesfatin-1 as a metabolic–stress integrator relevant to preconception health rather than a direct determinant of fertility (2, 8, 11, 12, 14, 27, 28).

Instead, nesfatin-1 may have greater utility when evaluated longitudinally or integrated into multi-parameter panels alongside metabolic, inflammatory, and reproductive markers. Such approaches may improve phenotypic stratification in women undergoing metabolic optimization prior to conception or assisted reproduction. Within this framework, nesfatin-1 is best considered a context-dependent endocrine indicator of systemic and reproductive physiology, providing a rationale for its investigation in assisted reproductive settings (2, 8, 11, 12, 14, 27, 28) (**Figure 2**).

## 4 Nesfatin-1 and assisted reproduction

ART, including in vitro fertilization (IVF) and intracytoplasmic sperm injection (ICSI), provide a clinically relevant model in which metabolic, endocrine, and stress-related factors converge to influence reproductive success (2, 8, 23). Ovarian stimulation, oocyte retrieval, embryo development, and implantation are highly sensitive to systemic metabolic status, inflammatory tone, and neuroendocrine signaling (8, 10, 12). Within this framework, nesfatin-1—given its role in integrating metabolic and stress-related pathways—represents a biologically plausible, yet insufficiently characterized, modulator of ART outcomes (2, 10, 30). Importantly, its potential role is unlikely to be deterministic; rather, nesfatin-1 may reflect the broader endocrine context in which ART procedures occur.

### 4.1 Potential central and systemic influences during ART

ART cycles are associated with substantial hormonal perturbations and psychological stress, both of which may influence neuroendocrine regulation. Central nesfatin-1 signaling interacts with corticotropin-releasing hormone and monoaminergic pathways and is activated under stress conditions, linking it to hypothalamic–pituitary–adrenal (HPA) axis activity (1, 12). This raises the possibility that ART-related psychological and physiological stressors may modulate circulating nesfatin-1 levels, thereby reflecting the neuroendocrine milieu during ovarian stimulation and early implantation.

Although direct evidence in ART populations remains limited, alterations in circulating nesfatin-1 observed in stress-related and metabolic conditions support its role as a responsive endocrine signal rather than a static biomarker (31–33). Systemically, nesfatin-1 is closely linked to insulin sensitivity and glucose homeostasis, both of which are critical determinants of ART success. Insulin resistance and metabolic dysfunction impair ovarian response, oocyte quality, and endometrial receptivity, particularly in women with obesity or polycystic ovary syndrome (PCOS) undergoing IVF (19, 34). In this context, nesfatin-1 may function as an indicator of metabolic–stress integration, capturing systemic conditions that influence reproductive competence rather than acting as a direct mediator of ART outcomes.

Endometrial receptivity and implantation are highly sensitive to metabolic and inflammatory signaling, including insulin sensitivity, cytokine activity, and vascular remodeling, all of which are critical for successful embryo implantation (8, 9, 23). Disruption of these pathways, particularly in conditions such as obesity and insulin resistance, is associated with impaired endometrial function and reduced implantation rates in ART (19, 34). Given its role in integrating metabolic, stress-related, and inflammatory signals, nesfatin-1 may indirectly influence endometrial receptivity through modulation of these pathways, although direct human evidence remains limited (2, 8, 23). Within this context, nesfatin-1 is more likely to reflect the systemic and local endocrine environment supporting implantation rather than acting as a direct determinant of endometrial competence.

### 4.2 Local ovarian and follicular considerations

In addition to systemic effects, nesfatin-1 is expressed in ovarian tissue and has been shown to modulate steroidogenesis, cellular proliferation, and apoptosis in granulosa and luteal cells (14, 32). These findings provide mechanistic support for a potential role of nesfatin-1 within the follicular microenvironment, where it may influence oocyte maturation and competence through local autocrine or paracrine signaling pathways (14, 28). Such local actions are particularly relevant in the context of controlled ovarian stimulation, where subtle alterations in the follicular environment can impact oocyte quality and downstream embryonic development.

However, human data on nesfatin-1 in follicular fluid during ART remain extremely limited. Existing studies have not systematically evaluated associations between nesfatin-1 and key clinical endpoints, including oocyte quality, fertilization rates, embryo development, or implantation success (2, 8, 35). This contrasts with other metabolic hormones, such as leptin and insulin, which have been more extensively studied and linked to ovarian response and ART outcomes. The absence of robust nesfatin-1 data in follicular fluid should therefore be interpreted as a critical knowledge gap rather than evidence against biological relevance (35, 36).

### 4.3 Interpretation and research gaps in ART settings

Taken together, current evidence supports a biologically plausible but largely untested role for nesfatin-1 in assisted reproduction. Its involvement in central stress signaling, systemic metabolic regulation, and local ovarian function suggests that nesfatin-1 may act as a context-dependent integrator of pathways known to influence ART success (2, 8, 28, 35). However, the absence of well-designed human studies limits the ability to draw causal or predictive conclusions.

Importantly, variability in ART protocols, stimulation regimens, metabolic phenotypes, and psychological stress levels introduces substantial heterogeneity, which is likely to influence nesfatin-1 dynamics and complicate interpretation (2, 12, 19). These factors underscore the need to consider nesfatin-1 within a broader physiological and clinical context rather than as a standalone biomarker.

Future research should prioritize well-characterized ART cohorts with standardized sampling of maternal plasma and, where feasible, follicular fluid, combined with detailed metabolic and reproductive outcome measures (2, 23, 35). Longitudinal study designs spanning ovarian stimulation, oocyte retrieval, and early pregnancy are particularly important to distinguish between stress-related, metabolic, and local ovarian contributions to nesfatin-1 regulation (2, 12, 23). Within the conceptual framework presented in **Figure 1**, nesfatin-1 is best interpreted as a context-sensitive endocrine indicator reflecting systemic and local conditions influencing ART outcomes, rather than a direct predictor of treatment success (2, 8, 22).

## 5 Pregnancy adaptations and complications

Pregnancy is characterized by profound and dynamic endocrine, metabolic, and inflammatory adaptations required to support fetal growth and prepare the mother for parturition and lactation (2, 20). These changes include progressive insulin resistance, alterations in lipid metabolism, activation of stress-response pathways, and tightly regulated immune modulation (2, 9, 12). At the maternal–fetal interface, the placenta plays a central role in integrating maternal metabolic, endocrine, and inflammatory signals, thereby coordinating nutrient transfer, hormonal regulation, and fetal development. Dysregulation of these adaptive processes contributes to common pregnancy complications, most notably gestational diabetes mellitus (GDM) and hypertensive disorders of pregnancy (2, 21, 37). Given its integrative role in metabolic, stress-related, and inflammatory signaling, nesfatin-1 has emerged as a candidate mediator within this interconnected regulatory network (2, 8, 23).

### 5.1 Gestational diabetes mellitus

GDM is characterized by glucose intolerance with onset or first recognition during pregnancy and reflects an inability to adequately compensate for pregnancy-induced insulin resistance. Nesfatin-1 has been implicated in glucose homeostasis through both central and peripheral mechanisms, including modulation of insulin secretion, insulin sensitivity, and hepatic glucose production (1, 38, 39). These properties provide a biological rationale for investigating nesfatin-1 in the context of GDM, where metabolic regulation is central to disease pathophysiology.

Human studies examining circulating nesfatin-1 levels in women with GDM report heterogeneous findings. Some studies describe elevated maternal plasma nesfatin-1 levels, potentially reflecting a compensatory response to insulin resistance, whereas others report reduced or unchanged levels compared with normoglycemic pregnancies (17, 20, 37). Rather than indicating inconsistency alone, this variability likely reflects the context-dependent regulation of nesfatin-1 across different metabolic states. Differences in diagnostic criteria, gestational timing of sampling, obesity status, and treatment exposure further contribute to inter-study heterogeneity (2, 19, 37). In addition, nesfatin-1 levels may vary dynamically across gestation, limiting the interpretability of single time-point measurements (2, 23, 40).

Beyond maternal circulation, nesfatin-1 has been detected in amniotic fluid and cord blood, suggesting a role in the intrauterine metabolic environment and potential fetal exposure (17, 24). At the maternal–fetal interface, alterations in nesfatin-1 may reflect interactions between maternal metabolic status, placental endocrine function, and fetal adaptive responses. However, the relative contribution of maternal, placental, and fetal sources remains unclear. Within the conceptual framework presented in **Figure 1**, changes in nesfatin-1 in GDM are therefore best interpreted as context-dependent indicators of metabolic stress and adaptation rather than disease-specific signals (37).

### 5.2 Hypertensive disorders of pregnancy

Hypertensive disorders of pregnancy, including gestational hypertension and preeclampsia, are complex conditions characterized by abnormal placentation, endothelial dysfunction, inflammation, and exaggerated stress and autonomic responses. Nesfatin-1 has been shown to influence sympathetic activity and cardiovascular regulation, providing a mechanistic basis for its potential involvement in these disorders (1, 41, 42).

Clinical studies investigating nesfatin-1 in hypertensive pregnancies are limited and heterogeneous (2, 22). Some reports suggest altered circulating nesfatin-1 levels in women with preeclampsia or gestational hypertension, whereas others report no significant differences compared with normotensive controls (2, 23). As observed in GDM, variability in study design, gestational age at sampling, disease severity, and coexisting metabolic conditions likely underlies these inconsistent findings (2, 37). Moreover, stress-related activation of the hypothalamic–pituitary–adrenal axis and systemic inflammation—both key features of hypertensive disorders—may independently modulate nesfatin-1 expression, further complicating interpretation (2, 9, 12).

Mechanistically, the potential role of nesfatin-1 in hypertensive disorders may be linked to its involvement in autonomic regulation, vascular tone, and inflammatory signaling rather than direct effects on blood pressure alone. At the placental level, impaired trophoblast invasion, altered vascular remodeling, and inflammatory activation are central features of disease pathogenesis. Nesfatin-1 may contribute indirectly to these processes by reflecting or modulating the broader metabolic–inflammatory environment, although direct placental mechanisms remain insufficiently characterized.

### 5.3 Interpretation across pregnancy complications

Taken together, current evidence does not support a uniform direction of change in nesfatin-1 across pregnancy complications. Instead, alterations in nesfatin-1 appear to reflect the balance between compensatory endocrine responses and maladaptive metabolic or inflammatory states (2, 21, 37). Differences in biological compartment (maternal plasma versus amniotic fluid), gestational timing, and underlying maternal phenotype are critical determinants of observed associations (2, 9, 23).

From a translational perspective, nesfatin-1 is unlikely to function as a standalone diagnostic biomarker for GDM or hypertensive disorders of pregnancy (17, 21). Rather, its value may lie in its ability to capture integrated metabolic and stress-related signaling when interpreted in combination with established biochemical and clinical parameters or when assessed longitudinally across gestation (2, 19, 23). This perspective is consistent with a model in which nesfatin-1 acts as a context-dependent endocrine integrator of maternal and placental physiology, rather than a unidirectional disease marker.

These considerations underscore the importance of compartment-specific and stage-specific interpretation and provide a rationale for further investigation of nesfatin-1 at the maternal–fetal interface. Taken together, available evidence suggests that nesfatin-1 integrates central stress and peripheral metabolic cues to modulate reproductive competence, rather than acting directly at a single level of the HPG axis (2, 8, 11, 12).

## 6 The maternal–fetal interface: fetal environment and development

The maternal–fetal interface represents a critical biological nexus through which maternal metabolic, endocrine, and inflammatory signals influence fetal growth and long-term developmental programming (2, 23, 24). During pregnancy, the placenta and amniotic compartment mediate bidirectional communication between mother and fetus, integrating nutrient supply, hormonal signaling, and immune tolerance (8, 17). Peptides involved in energy and stress regulation, including nesfatin-1, are increasingly recognized as potential contributors to this intrauterine signaling environment (2, 8, 17).

### 6.1 Nesfatin-1 at the maternal–fetal interface

Nesfatin-1 has been detected in biological compartments directly related to the fetal environment, including amniotic fluid and umbilical cord blood, indicating that the developing fetus is exposed to nesfatin-1 during gestation (17, 24). While the precise origin of amniotic nesfatin-1 remains unclear, potential sources include maternal circulation via placental transfer, placental synthesis, and fetal production. The relative contribution of these sources likely varies across gestation and maternal metabolic context.

Importantly, amniotic fluid does not represent a simple filtrate of maternal plasma. Instead, it reflects the integrated intrauterine milieu, incorporating fetal urine, pulmonary secretions, placental transport, and inflammatory mediators. Consequently, nesfatin-1 levels in amniotic fluid may provide information distinct from maternal circulating concentrations and may better capture intrauterine metabolic or inflammatory conditions (17). This compartmental specificity is critical when interpreting associations between nesfatin-1 and fetal outcomes.

### 6.2 Associations with fetal growth and metabolic programming

Emerging human data suggest associations between nesfatin-1 levels and fetal growth parameters, although findings remain limited and heterogeneous. Studies measuring nesfatin-1 in cord blood have reported correlations with birth weight, neonatal adiposity, and metabolic markers, raising the possibility that nesfatin-1 participates in fetal energy homeostasis or appetite-related programming (16,v38). However, the direction and strength of these associations vary across studies and are influenced by maternal metabolic status, including obesity and gestational diabetes mellitus.

From a developmental programming perspective (32, 33), nesfatin-1 is of particular interest because of its established roles in appetite regulation, glucose metabolism, and stress responsiveness in postnatal life. In animal models, early-life exposure to metabolic and stress-related signals shapes hypothalamic circuitry and long-term energy balance, suggesting plausible mechanisms by which intrauterine nesfatin-1 exposure could influence offspring phenotype (1). In humans, however, direct causal evidence linking fetal nesfatin-1 exposure to long-term metabolic outcomes is currently lacking.

### 6.3 Interpretation within a context-dependent framework

Alterations in nesfatin-1 at the maternal–fetal interface should be interpreted within the broader metabolic and inflammatory context of pregnancy. Conditions such as gestational diabetes mellitus, hypertensive disorders, and maternal obesity are characterized by insulin resistance, low-grade inflammation, and stress-axis activation, all of which may independently modulate nesfatin-1 expression and transport (9, 23). As a result, elevated or reduced nesfatin-1 levels in amniotic fluid or cord blood may reflect adaptive or compensatory responses rather than direct pathogenic mechanisms (2, 17, 21, 24).

Within the conceptual framework outlined in **Figure 1**, nesfatin-1 at the maternal–fetal interface can be viewed as a marker of intrauterine metabolic–endocrine state rather than a fetal-specific hormone. This distinction is essential to avoid overinterpretation of cross-sectional associations and to guide the design of future studies.

### 6.4 Knowledge gaps and future directions

Despite growing interest, significant gaps remain in our understanding of nesfatin-1 at the maternal–fetal interface. Longitudinal studies assessing nesfatin-1 across pregnancy, alongside detailed maternal metabolic phenotyping and placental characterization, are lacking. The extent of placental synthesis and transport of nesfatin-1 has not been definitively established, and standardized methods for measuring nesfatin-1 in amniotic fluid are not yet available.

**Figure 3:**
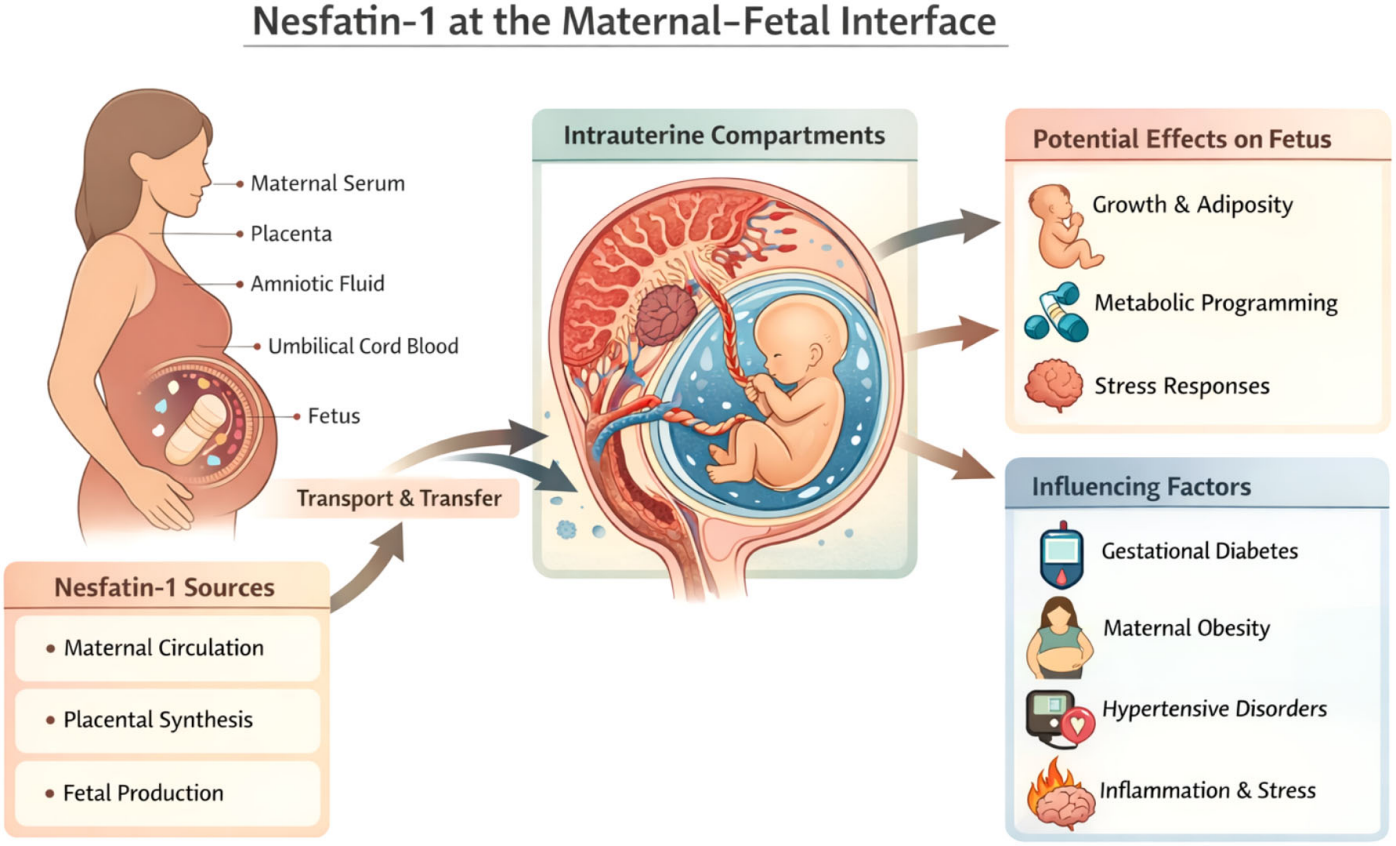
Nesfatin-1 at the maternal–fetal interface: sources, compartments, and potential developmental implications. Nesfatin-1, derived from nucleobindin-2 (NUCB2), is detectable in multiple biological compartments relevant to the fetal environment, including maternal circulation, placenta, amniotic fluid, and umbilical cord blood. Potential sources of nesfatin-1 at the maternal–fetal interface include maternal peripheral tissues, placental synthesis, and fetal production, although their relative contributions remain incompletely defined. Amniotic fluid represents an integrated intrauterine compartment reflecting maternal metabolic and inflammatory status, placental transport, and fetal secretions rather than a direct proxy of maternal plasma levels. Alterations in nesfatin-1 within these compartments may be influenced by pregnancy-related conditions such as gestational diabetes mellitus, maternal obesity, hypertensive disorders of pregnancy, and systemic inflammation or stress. Through exposure during gestation, nesfatin-1 may participate in fetal growth regulation and early metabolic and stress-related programming; however, causal relationships in humans remain to be established. Solid arrows indicate established or supported pathways, while dashed arrows represent hypothesized or context-dependent mechanisms (14, 17, 24, 39, 42, 43).

Future research should prioritize integrative designs combining maternal plasma, amniotic fluid, cord blood, and placental tissue analyses to clarify the sources, regulation, and functional relevance of nesfatin-1 in the fetal environment. Such approaches are essential to determine whether nesfatin-1 contributes to developmental programming or primarily reflects maternal metabolic and inflammatory conditions during pregnancy (**Figure 3**).

## 7 Lactation and postpartum life

The postpartum period represents a unique physiological transition during which maternal metabolism, endocrine signaling, and stress responsiveness are recalibrated following pregnancy. Lactation imposes substantial energetic demands and is accompanied by coordinated hormonal adaptations that support milk production while facilitating maternal metabolic recovery. In this context, nesfatin-1 has emerged as a potential mediator linking maternal metabolic state, lactational physiology, and early postnatal exposure of the infant (16).

### 7.1 Nesfatin-1 in human milk

Human studies have demonstrated the presence of nesfatin-1 in breast milk, including both colostrum and mature milk, establishing lactation as a distinct biological compartment for nesfatin-1 exposure in early life (13, 15, 24). The detection of nesfatin-1 in milk suggests active regulation rather than passive diffusion from maternal circulation, although the relative contribution of systemic versus mammary gland–specific synthesis remains unclear.

Reported milk nesfatin-1 concentrations vary widely across studies, likely reflecting differences in sampling time postpartum, maternal metabolic phenotype, assay methodology, and milk fraction analyzed. Similar to other metabolic hormones detected in human milk, nesfatin-1 levels may change dynamically during the early postpartum period as lactation is established and maternal energy balance shifts from pregnancy-associated insulin resistance toward metabolic normalization (15).

### 7.2 Maternal metabolic state and postpartum adaptation

The postpartum period is characterized by marked interindividual variability in metabolic recovery, influenced by pre-pregnancy body mass index, gestational weight gain, gestational diabetes mellitus, and breastfeeding intensity. Nesfatin-1 has been linked to glucose homeostasis, lipid metabolism, and inflammatory signaling in non-pregnant populations, suggesting that postpartum nesfatin-1 dynamics may reflect maternal metabolic adaptation following pregnancy (1, 2, 9).

In women with prior gestational diabetes or obesity, altered nesfatin-1 levels in the postpartum period may indicate persistent metabolic stress or incomplete recovery of insulin sensitivity (2, 37). However, human data directly linking postpartum nesfatin-1 trajectories to maternal metabolic outcomes remain sparse (2, 16, 18). Longitudinal studies tracking nesfatin-1 from late pregnancy through lactation are needed to clarify whether nesfatin-1 reflects adaptive lactational metabolism or residual pregnancy-associated metabolic dysregulation (2, 16, 18, 30).

### 7.3 Potential implications for infant development

The presence of nesfatin-1 in human milk raises the possibility that it contributes to early postnatal endocrine signaling influencing infant appetite regulation, energy balance, and stress responsiveness. Nesfatin-1 is known to regulate feeding behavior and metabolic processes in postnatal and adult models, supporting biological plausibility for such effects (1, 30, 43). In humans, however, direct evidence linking milk nesfatin-1 exposure to infant growth patterns or metabolic outcomes is currently limited.

Milk-borne hormones are thought to act locally within the gastrointestinal tract or indirectly through neuroendocrine pathways rather than entering the systemic circulation intact. Within this framework, nesfatin-1 may function as a signaling molecule shaping early feeding behavior or metabolic programming rather than as a classical circulating hormone in the infant. Importantly, the effects of milk nesfatin-1 are likely context-dependent and modulated by concurrent exposure to other milk-borne bioactive factors (16).

### 7.4 Interpretation and research priorities

From a translational perspective, nesfatin-1 in human milk should be viewed as a potential marker of maternal metabolic and endocrine state during lactation, as well as a candidate contributor to postnatal developmental signaling (16, 18). However, substantial methodological challenges complicate interpretation, including variability in milk collection protocols, lack of standardized reporting of lactation stage, and limited validation of assays for milk matrices (2, 18).

Future studies should prioritize standardized longitudinal designs incorporating maternal plasma and milk sampling, detailed metabolic phenotyping, and infant growth and metabolic outcomes. Such approaches are essential to determine whether nesfatin-1 plays an active role in postpartum metabolic adaptation and early-life programming or primarily reflects maternal physiological state during lactation (30, 44). Within the life-course framework presented in **Figure 1**, lactation represents a critical window in which nesfatin-1 may bridge maternal metabolic recovery and infant developmental trajectories.

## 8 Reconciling conflicting human biomarker data

Human studies assessing nesfatin-1 across reproduction, pregnancy, and early life report highly heterogeneous findings, with circulating or compartment-specific levels described as increased, decreased, or unchanged across similar clinical conditions. Rather than reflecting biological inconsistency, this variability is best understood as the result of context-dependent regulation combined with substantial methodological heterogeneity. Integrating these dimensions is essential for meaningful interpretation of nesfatin-1 as a reproductive and developmental biomarker.

### 8.1 Biological context: why directionality is not fixed

Nesfatin-1 is regulated by multiple, partially overlapping endocrine axes, including metabolic, stress-related, inflammatory, and reproductive pathways (1, 2). The relative contribution of these axes varies markedly across reproductive states.

During preconception and infertility, nesfatin-1 levels may reflect chronic metabolic imbalance or stress-related suppression of reproductive function rather than intrinsic ovarian pathology. In pregnancy, progressive insulin resistance, activation of the hypothalamic–pituitary–adrenal axis, and immune modulation create a dynamic endocrine milieu in which nesfatin-1 may increase as a compensatory response or decrease in states of endocrine exhaustion. Similarly, at the maternal–fetal interface and during lactation, nesfatin-1 levels likely reflect adaptive signaling rather than disease-specific effects (7, 15).

Importantly, nesfatin-1 does not behave as a permissive “on/off” signal analogous to leptin but rather as a stress- and context-sensitive integrator, responding to metabolic load, inflammation, and neuroendocrine activation. As a result, opposing directions of change may be biologically appropriate at different stages of disease progression or physiological adaptation (2, 12, 38).

### 8.2 Compartment specificity: plasma, amniotic fluid, and milk are not interchangeable

A major source of apparent inconsistency arises from the assumption that nesfatin-1 measured in different biological compartments reflects the same biological process. As illustrated in **Figures 1, 2**, nesfatin-1 in maternal plasma, amniotic fluid, cord blood, and human milk likely represents distinct biological signals.

Circulating nesfatin-1 reflects the integrated output of multiple peripheral tissues and does not reliably mirror central hypothalamic activity (2). Amniotic fluid nesfatin-1 reflects the intrauterine metabolic–inflammatory environment rather than maternal plasma concentrations alone, while milk nesfatin-1 represents a postnatal exposure compartment influenced by mammary gland regulation and maternal metabolic recovery. Failure to account for this compartmental specificity inevitably leads to conflicting interpretations.

### 8.3 Methodological heterogeneity across human studies

Methodological variability further amplifies biological heterogeneity. Commercial immunoassays used to measure nesfatin-1 differ in specificity, with variable cross-reactivity to NUCB2 fragments, and are rarely validated for complex matrices such as amniotic fluid or human milk (2). Preanalytical factors—including fasting status, circadian timing, gestational age, postpartum day, sample handling, and freeze–thaw cycles—are inconsistently reported and may substantially influence measured concentrations (**Table 1**). Although no universally standardized or validated reference assay for Nesfatin-1 exists, numerous studies have quantified its concentrations using commercially available immunoassays, primarily enzyme-linked immunosorbent assays (ELISA) -based methods. These assays vary in antibody specificity, calibration standards, and analytical sensitivity, leading to potential inter-study variability. Consequently, the absence of assay harmonization limits comparability across studies, and reported concentrations should be interpreted within the context of methodological differences.

**Table 1:** Reported nesfatin-1 concentrations in human biological tissues and fluids.

Biological compartment	Reported concentration range	Physiological context studied	Key notes for interpretation (with representative citations)
Maternal plasma/serum	~0.3–5.0 ng/mL	Healthy women, obesity, PCOS, GDM, hypertensive disorders, postpartum	High inter-study variability; influenced by BMI, insulin resistance, stress, gestational stage, and assay type. Plasma and serum are not interchangeable (2, 19, 20)
Follicular fluid	Limited data; comparable to or slightly higher than plasma	IVF/ICSI cycles (very few studies)	Human data are scarce; local ovarian contribution plausible but unconfirmed; no consistent links to ART outcomes (8, 14).
Amniotic fluid	~0.1–3.0 ng/mL	Second–third trimester pregnancies, obesity, GDM	Reflects integrated intrauterine metabolic–inflammatory milieu rather than maternal plasma alone; gestational age and maternal metabolic state are key modifiers (17).
Umbilical cord blood	~0.2–4.0 ng/mL	Term and preterm neonates, LGA/SGA, maternal metabolic disorders	May reflect fetal exposure and/or placental transfer; associations with birth weight reported but inconsistent (24, 39).
Placental tissue	Qualitative expression (protein/mRNA detected)	Normal and complicated pregnancies	Quantitative concentration data are limited; placental synthesis is plausible but not definitively quantified in humans (8).
Human milk – colostrum	~0.5–10 ng/mL	Early postpartum (days 1–5)	Typically higher than mature milk; influenced by maternal metabolic status and early postpartum adaptation (18).
Human milk – mature milk	~0.2–5.0 ng/mL	Established lactation	Declines over postpartum time; variability depends on milk fraction analyzed and assay methodology (2, 18).
Cerebrospinal fluid (CSF)	Low ng/mL range (limited human data)	Non-pregnant adults	Rarely measured in reproductive contexts; central nesfatin-1 does not directly correlate with circulating levels (2, 41).

CSF, cerebrospinal fluid; GDM, gestational diabetes mellitus; ICSI, intracytoplasmic sperm injection; IVF, *in vitro* fertilization; LGA, large for gestational age; PCOS, polycystic ovary syndrome; SGA, small for gestational age.

In reproductive and pregnancy studies, additional confounders include maternal body mass index, parity, medication use (e.g., insulin, metformin), mode of conception, and psychological stress, all of which may independently modulate nesfatin-1 levels (9, 40). Cross-sectional designs further limit interpretation by failing to capture dynamic endocrine trajectories across reproductive transitions.

## 9 Clinical and translational implications

The expanding body of human data reviewed here highlights nesfatin-1 as a biologically meaningful, yet complex, endocrine signal across reproduction and early life. While current evidence does not support nesfatin-1 as a standalone diagnostic biomarker, it does point to several translational contexts in which nesfatin-1 measurement may provide clinically relevant information when interpreted appropriately (2, 8, 20).

### 9.1 Nesfatin-1 as a stratification and context marker

Across infertility, pregnancy complications, and postpartum life, nesfatin-1 appears to reflect the integrated metabolic–stress–inflammatory state rather than a single pathological process (1, 2). In this respect, nesfatin-1 may be most useful as a stratification biomarker, identifying subgroups of women with distinct metabolic or stress-related phenotypes within broader clinical diagnoses such as polycystic ovary syndrome, gestational diabetes mellitus, or hypertensive disorders of pregnancy (19, 20). For example, divergent nesfatin-1 levels among women with similar glycemic profiles may reflect differences in stress-axis activation or inflammatory burden, factors known to influence reproductive and pregnancy outcomes (12, 23). Incorporating nesfatin-1 into multi-parameter panels alongside insulin resistance indices, inflammatory markers, and reproductive hormones may therefore improve phenotypic resolution without relying on nesfatin-1 as a single determinant (2, 22).

### 9.2 Longitudinal assessment across reproductive transitions

A key translational insight emerging from this review is the limitation of cross-sectional nesfatin-1 measurements. Given its dynamic regulation, nesfatin-1 is more likely to be informative when assessed longitudinally, capturing trajectories across critical reproductive transitions such as preconception optimization, early to late pregnancy, and postpartum metabolic recovery (2).

In pregnancy, longitudinal nesfatin-1 profiling may help distinguish adaptive endocrine responses from maladaptive patterns associated with persistent insulin resistance or inflammatory stress (2, 9, 23). Similarly, postpartum nesfatin-1 trajectories could provide insight into maternal metabolic recovery following gestational diabetes or obesity, particularly in the context of lactation, which itself represents a major metabolic intervention (18, 30).

### 9.3 Implications for assisted reproduction and pregnancy care

Although direct evidence linking nesfatin-1 to assisted reproductive technology outcomes is limited, its established roles in metabolic regulation and stress responsiveness suggest potential relevance in ART settings characterized by hormonal perturbation and psychological stress (1, 12, 23). In this context, nesfatin-1 measurement may offer indirect insight into systemic conditions influencing ovarian response or implantation rather than serving as a predictor of cycle success (21, 22, 37).

In pregnancy care, nesfatin-1 is unlikely to replace established screening tools for gestational diabetes or hypertensive disorders. However, it may contribute to risk stratification or monitoring when integrated with clinical and biochemical parameters, particularly in research settings aimed at understanding heterogeneity within these conditions (2, 21, 37).

### 9.4 Methodological and regulatory considerations

From a translational perspective, several barriers must be addressed before nesfatin-1 measurement can be meaningfully incorporated into clinical research or practice. These include the lack of standardized, peptide-specific assays, limited validation in complex matrices such as amniotic fluid and human milk, and absence of reference ranges stratified by sex, gestational stage, and metabolic phenotype (2, 22).

Furthermore, the absence of a definitively identified receptor limits mechanistic interpretation and complicates causal inference. Until receptor identity and signaling specificity are clarified, nesfatin-1 should be regarded as an indicator of endocrine context rather than a direct therapeutic target (2, 25, 26).

### 9.5 Implications for future interventional studies

Despite these limitations, nesfatin-1 remains a promising candidate for inclusion in mechanistic and interventional studies addressing metabolic and reproductive health (1, 2, 8). Weight loss interventions, stress-reduction programs, lifestyle modification, and pharmacological treatments that alter insulin sensitivity or inflammatory tone may all influence nesfatin-1 dynamics (34, 39). Assessing nesfatin-1 responses to such interventions could help clarify its role as a responsive biomarker and its potential contribution to reproductive and metabolic adaptation (2, 23). Within this framework, nesfatin-1 is best positioned not as a diagnostic endpoint, but as a research biomarker that captures adaptive endocrine signaling across the reproductive and early-life continuum (19, 20).

## 10 Knowledge gaps and future directions

Despite growing interest in nesfatin-1 as an endocrine signal linking metabolism, stress, and reproduction, substantial gaps remain in our understanding of its physiological role and translational relevance across human reproduction and early life. Addressing these gaps is essential to move the field beyond descriptive associations toward mechanistic insight and clinical utility.

### 10.1 Incomplete understanding of sources and regulation

A fundamental knowledge gap concerns the relative contribution of maternal, placental, and fetal sources to nesfatin-1 levels in different biological compartments. While nesfatin-1 has been detected in maternal plasma, amniotic fluid, cord blood, and human milk, the extent to which these pools reflect local synthesis versus transport remains unclear (16, 17, 24). In particular, quantitative data on placental expression and regulated transport of nesfatin-1 in humans are limited (23, 40).

Future studies combining maternal circulation, placental tissue, amniotic fluid, and neonatal samples within the same cohort are required to delineate compartment-specific regulation. Such integrative designs would clarify whether nesfatin-1 primarily reflects maternal endocrine state or contributes directly to fetal signaling (17, 21, 24).

### 10.2 Lack of longitudinal human studies

Most available human studies assess nesfatin-1 at a single time point, limiting interpretation in dynamic physiological states such as pregnancy and lactation. Given the context-dependent regulation of nesfatin-1, longitudinal profiling across reproductive transitions—from preconception through pregnancy and into the postpartum period—is a critical unmet need (2, 20, 37).

Longitudinal designs would allow differentiation between adaptive endocrine responses and maladaptive trajectories associated with persistent metabolic stress, such as progression from gestational diabetes to postpartum dysglycemia (2, 23). Such approaches are particularly relevant for understanding nesfatin-1 dynamics during lactation and maternal metabolic recovery (22, 37).

### 10.3 Methodological standardization and assay validation

Methodological heterogeneity remains a major barrier to progress. Commercial immunoassays vary in specificity and are rarely validated for complex matrices such as amniotic fluid or human milk. Standardized protocols for sample collection, processing, storage, and reporting are lacking, complicating cross-study comparisons (2, 22, 23). Future work should prioritize assay validation studies, including assessment of cross-reactivity with NUCB2 fragments, matrix effects, and intra- and inter-assay variability (23, 25). Establishing consensus reporting standards for nesfatin-1 studies in reproductive and perinatal contexts would substantially enhance reproducibility and interpretability (19, 20, 22).

### 10.4 Unresolved receptor identity and signaling mechanisms

The absence of a definitively identified nesfatin-1 receptor represents a critical mechanistic gap. Without receptor de-orphanization, it remains difficult to link circulating or compartment-specific nesfatin-1 levels to downstream signaling activity or biological effects (1). This limitation constrains causal inference and hampers therapeutic exploration.

Advances in receptor identification and signaling pathway characterization are essential to determine whether nesfatin-1 functions primarily as an active effector hormone, a modulator of other endocrine systems, or a marker of adaptive stress responses in reproductive physiology (25, 31).

### 10.5 Integration into multi-marker and interventional studies

Given its pleiotropic regulation, nesfatin-1 is unlikely to be informative in isolation. Future studies should integrate nesfatin-1 into multi-marker panels including metabolic, inflammatory, and reproductive hormones to better capture endocrine context. In addition, interventional studies—such as lifestyle modification, weight loss, stress reduction, or pharmacological treatment—offer opportunities to examine nesfatin-1 responsiveness and clarify its role as a dynamic biomarker (9).

Such approaches are particularly relevant in assisted reproduction, pregnancy complication risk stratification, and postpartum metabolic follow-up, where understanding adaptive versus maladaptive endocrine signaling is clinically meaningful.

## 11 Conclusion

Accumulating human evidence positions nesfatin-1 as a biologically meaningful yet context-dependent endocrine signal operating across the continuum of human reproduction and early life. Rather than functioning as a unidirectional or permissive regulator, nesfatin-1 appears to integrate metabolic status, stress-axis activity, inflammatory tone, and reproductive signaling, particularly during physiologically dynamic states such as preconception, pregnancy, lactation, and the postpartum period (**Figure 1**).

Across infertility, assisted reproduction, pregnancy complications, and early developmental contexts, reported nesfatin-1 levels are highly heterogeneous. This variability likely reflects compartment-specific regulation and sensitivity to biological context rather than inconsistent biology. Importantly, circulating nesfatin-1 does not directly mirror central neuroendocrine activity, and measurements obtained from maternal plasma, amniotic fluid, cord blood, or human milk represent distinct and non-interchangeable biological signals (**Figures 1**-**3**).

From a translational perspective, current evidence does not support nesfatin-1 as a standalone diagnostic biomarker or therapeutic target. However, when interpreted longitudinally and in combination with metabolic, inflammatory, and reproductive markers, nesfatin-1 may provide complementary insight into endocrine adaptation, phenotypic heterogeneity, and maternal–fetal metabolic context (**Figures 2**, **3**). Its greatest value therefore lies in stratification and research-oriented applications rather than isolated cross-sectional assessment.

Substantial knowledge gaps remain, including limited quantitative data on placental expression and transport in humans, unresolved receptor identity, and the lack of standardized, peptide-specific assays validated for complex biological matrices. Addressing these gaps through integrative, longitudinal study designs spanning maternal, placental, fetal, and postnatal compartments will be essential to clarify whether nesfatin-1 primarily reflects maternal endocrine state or contributes directly to fetal and postnatal signaling (**Figure 3**).

In summary, framing nesfatin-1 within a life-course and compartment-based perspective provides a coherent framework to reconcile heterogeneous human data and to guide future mechanistic and translational research into metabolic, stress-related, and reproductive adaptation across early life.
